# HIV-positive pregnant and postpartum women's perspectives about Option B+ in Malawi: a qualitative study

**DOI:** 10.7448/IAS.19.1.20919

**Published:** 2016-06-15

**Authors:** Leila Katirayi, Hazel Namadingo, Mafayo Phiri, Emily A Bobrow, Allan Ahimbisibwe, Aida Yemane Berhan, Nicole Buono, Karen Marie Moland, Thorkild Tylleskär

**Affiliations:** 1 Elizabeth Glaser Pediatric AIDS Foundation, Washington, DC, USA; 2 Elizabeth Glaser Pediatric AIDS Foundation, Lilongwe, Malawi; 3 Center for International Health, University of Bergen, Bergen, Norway

**Keywords:** Option B+, lifelong HIV treatment, counselling, same-day initiation, prevention of mother-to-child transmission of HIV, qualitative

## Abstract

**Introduction:**

The implementation of lifelong antiretroviral treatment (ART) for all pregnant women (Option B+) in Malawi has resulted in a significant increase in the number of HIV-positive pregnant women initiating treatment. However, research has highlighted the challenge of retaining newly initiated women in care. This study explores barriers and facilitators that affect a woman's decision to initiate and to adhere to Option B+.

**Methods:**

A total of 39 in-depth interviews and 16 focus group discussions were conducted. Eligible women were ≥18 years old, living with HIV and either pregnant and receiving antenatal care from a study site or had delivered a child within the last 18 months, breastfed their child and received services at one of the study sites. Eligible women were identified by healthcare workers (HCWs) in the antenatal clinic and ART unit. Focus groups were also conducted with HCWs employed in these departments. Qualitative data were analyzed using Maxqda version 10 (VERBI Software, Berlin, Germany).

**Results:**

The general perception towards the drug regimen used in Option B+ was positive; women reported fewer side effects and acknowledged the positive benefits of ART. Women felt hopeful about prolonging their life and having an HIV-uninfected baby, yet grappled with the fact that ART is a lifelong commitment. Women and HCWs discussed challenges with the counselling services for prevention of mother-to-child HIV transmission under the new Option B+ guidelines, and many women struggled with initiating ART on the same day as learning their HIV status. Women wanted to discuss their circumstances with their husbands first, receive a CD4 count and obtain an HIV test at another facility to confirm their HIV status. HCWs expressed concern that women might just agree to take the drugs to please them. HCWs also discussed concerns around loss to follow-up and drug resistance.

**Conclusions:**

Although Option B+ has significantly increased the number of women initiating ART, there are still challenges that need to be addressed to strengthen initiation, adherence and retention in care. Strategies to strengthen the counselling services upon diagnosis need to be developed to improve same-day initiation of ART and long-term adherence.

## Introduction

Prevention of mother-to-child transmission (PMTCT) involves a cascade of services provided to HIV-positive women including antenatal services and HIV testing during pregnancy, use of antiretroviral treatment (ART), safe childbirth practices, appropriate infant feeding and testing the child for HIV. In July 2011, Malawi initiated Option B+, an adaptation of the WHO 2010 PMTCT guidelines [1]. Under this new approach, all HIV-positive pregnant and breastfeeding women initiate ART for life, irrespective of their CD4 count or clinical staging (Table 1). Option B+ services were decentralized to primary care facilities and integrated with maternal and child health (MCH) services, and nurses were trained to initiate women on ART [2].

**Table 1:** Differences in PMTCT before and after Option B+ in Malawi

	Before Option B+	After Option B+
CD4 test	CD4 count test was required.	No CD4 count test required.
Initiation of ART	Lifelong ART only initiated if woman had a CD4 count <350 cells/µL.	Initiate women on lifelong ART regardless of CD4 count.
Time to initiation of ART from learning HIV status	Women initiated within 2 to 6 weeks of learning HIV status.	Women initiated on the same day they learn their HIV status.
Number of counselling sessions received before initiating ART	ART was initiated at the third counselling session.	Initiate ART at the first counselling session.
Drug regimen for ART-ineligible women	AZT during pregnancy; AZT/3TC and single dose NVP at delivery.	TDF/3TC/EFV.
Drug regimen for ART-eligible women	AZT/3TC/NVP during pregnancy and delivery.	TDF/3TC/EFV.
Frequency of medication	Medicine taken twice a day.	Medicine taken once a day.
“Treatment supporter” requirement	Women required to have a treatment supporter present when initiating ART.	No treatment supporter required.
Where initiation occurs	Possible to initiate in ART clinic only.	Can initiate in ANC, ART, MCH and labour and delivery.
Postpartum management	Women with CD4 <350 cells/µL continued lifelong ART; women with CD4 count ≥350 cells/µL received AZT/3TC for seven days after delivery.	Lifelong ART is continued in all women regardless of CD4 cell count.
Infant antiretroviral prophylaxis	Mother received AZT: Single-dose NVP plus AZT for seven days to four weeks after birth (depending on whether maternal AZT was given for at least four weeks or less than four weeks antepartum). Mother received ART: AZT for seven days.	NVP for six weeks after birth.
Breastfeeding guidance	Women told to exclusively breastfeed for six months and then stop breastfeeding.*	Women encouraged to exclusively breastfeed for six months and then introduce complementary feedings along with continued breastfeeding for up to two years postpartum.*

* The change in breastfeeding guidelines was not part of Option B+; however, the shift in breastfeeding recommendations in Malawi occurred at the same time as Option B+. ANC, antenatal clinic; ART, antiretroviral therapy; AZT, zidovudine; 3TC, lamivudine; MCH, maternal child health clinic; NVP, nevirapine; PMTCT, prevention of mother-to-child transmission; TDF, tenofovir.

Another shift in ART initiation in Malawi under the Option B+ guidelines was to initiate women on ART the same day they receive their HIV-positive diagnosis. Before receiving their first supply of ART, women receive general group counselling, followed by individual counselling. Women receive monthly adherence counselling for the next six months followed by a general adherence counselling session to transition to three-month follow-up visits. Targeted counselling is provided any time a provider notices that there are adherence challenges. Initiating all women on lifelong ART the same day they learn their HIV status resulted in a sevenfold increase in the number of pregnant and breastfeeding women starting ART, from 1257 in the second quarter of 2011 to 10,663 in the third quarter of 2012 [3].

Despite the increased number of pregnant women initiated on ART, there have been challenges with the roll-out of Option B+ in Malawi. A study on retention in care in Malawi showed that six months after ART initiation, the proportion of Option B+ patients who had had no follow-up visit after ART initiation or who were lost to follow-up (LTFU) was 23.9% (95% CI: 22.6 to 25.3%) [4]. The same study reported that Option B+ patients who started ART while pregnant were five times more likely to fail to return to the clinics after the initial visit compared to patients who started ART for their own health. Previous research on Option B+ in Malawi cited transportation barriers, not understanding the initial ART counselling session and ART side effects as some of the main reasons women do not return to the facility [5]. However, the majority of research on Option B+ has been quantitative, not allowing for in-depth exploration of women's perspectives towards Option B+.

More knowledge is needed about what shapes acceptance of and adherence to ART among women initiating ART during pregnancy. An exploration of these questions has been identified as a priority in Malawi and elsewhere [6]. In particular, there is an urgent need to understand why a large proportion of women enrolled in the Option B+ programme do not return after the initial visit. Therefore, this study was designed to explore the critical issues shaping acceptance to initiate ART and adherence to ART among women under Option B+.

## Methods

### Study design

This qualitative study used in-depth interviews (IDIs) and focus group discussions (FGDs) to explore the experiences and perceptions of pregnant and postpartum HIV-positive women enrolled in Option B+, and FGDs to explore the perspectives of healthcare workers (HCWs). Study participants only participated in either an IDI or an FGD, but not both; data were only collected at one time point.

### Site selection

To gain perspectives from the different types of settings, data were collected from Likuni Mission Hospital (urban), Kabadula Community Hospital (rural) and Dedza and Mchinji District Hospitals (both peri-urban). Sites were selected that were either government-owned (free services) or private (pay for services) to gain perspectives from both types of sites. Within these parameters, the sites with the highest volume of HIV-positive pregnant women were selected.

### Data collection

Data were collected between September and December 2013 by trained research assistants (RAs) using semi-structured interviews (IDIs) and FGDs that were audio-recorded. The interview and topic guides included questions about knowledge of HIV/AIDS, perceived risks and benefits of lifelong ART, community perceptions about HIV and ART, and perceptions and experiences related to the ability of the health facility to provide the needed services. The interviews were conducted by one RA while the FGD was conducted by two RAs; one moderated, while the other observed and took notes. On average, interviews took 60 minutes and FGDs lasted 130 minutes. Both IDIs and FGDs were conducted in the local language of Chichewa by native speakers.

Eligible women were 18 years of age or older, living with HIV and either a) pregnant and receiving antenatal care (ANC) from a study site or b) had delivered a live child within the last 18 months who was still alive, breastfed their child and received services at one of study sites. Only women who initiated the drug regimen associated with Option B+ (tenofovir/lamivudine/efavirenz) within the recent pregnancy were included. For some of the women, this was their first experience with HIV medication, while other women had been enrolled in a PMTCT programme during previous pregnancies but were not currently receiving ART at entrance into ANC.

HCWs providing services in the ANC and ART units were trained on eligibility and would refer eligible women to the RAs on site. To determine if a woman was on Option B+, HCWs would verify that they were receiving the drug regimen associated with Option B+. Once the RAs confirmed eligibility, they would obtain informed consent and either conduct an IDI or invite the women to return on a set day for a FGD. Women who arrived earlier in the day were offered participation in the IDI and those who arrived later were invited to return for the FGD. Late arrival at the facility did not allow women time to participate in an IDI and obtain public transport home.

The only eligibility requirement for HCWs was to have worked in the ANC or ART departments of the selected facilities for the previous six months. HCWs were identified and referred to the RAs by the nurse in charge. RAs confirmed eligibility and invited eligible HCWs to participate in a FGD on a set day. All eligible HCWs were invited to participate, and this number never exceeded 12 HCWs within any health unit.

All study participants provided written informed consent. The study received ethical approval from the Malawi Ministry of Health National Health Sciences Research Committee.

### Analysis

The recorded IDIs and FGDs were simultaneously transcribed and translated verbatim from Chichewa into English by the RAs. Data were analyzed using thematic analysis [7]. A code list was generated by the principal investigator and co-investigator based on the study objectives and findings. Transcripts were uploaded into the qualitative analysis software Maxqda version 10 (VERBI Software, Berlin, Germany) and coded by three individuals. At three time points throughout the coding process, transcripts were compared and examined line by line to ensure consistency in the application of codes between the different coders. The code list was then updated and code definitions further defined. Data were summarized through descriptive, text-based summaries and data display matrices. In the data matrices, data were separated by study populations and compared for similarities and differences. The summaries and data matrices helped to identify the overarching themes and categories. Quotes were selected that were representative of the main themes.

## Results

Thirty-nine IDIs were conducted with women enrolled in the Option B+ programme, including 19 pregnant women and 20 postpartum women. Sixteen FGDs were conducted; four with HCWs, eight with postpartum women and four with pregnant women. Across all groups, the majority had primary education and were married. There were some differences in age across the groups: the pregnant women in the IDIs were older, with an average age of 31 to 35 years, whereas the pregnant women in the FGDs were primarily aged 26 to 35 years. In the postpartum group, women tended to be slightly younger, with those participating in the IDIs primarily aged 26 to 30 years old (Table 2). The majority of HCWs were nurses with 1-10 years of experience and the greater proportion were between the ages of 25-34 (Table 3).

**Table 2:** Overview of pregnant and postpartum women

	Individual interviews		Focus group discussions	
		
	Pregnant women *n*=19 *n* (%)	Postpartum women *n*=20 *n* (%)	Pregnant women (four FGDs) *n*=19 *n* (%)	Postpartum women (eight FGDs) *n*=74 *n* (%)
Age (years)				
15 to 20	1 (5)	1 (5)	1 (5)	4 (5)
21 to 25	4 (21)	7 (35)	4 (21)	20 (27)
26 to 30	3 (16)	7 (35)	6 (32)	22 (30)
31 to 35	10 (53)	5 (25)	6 (32)	18 (24)
36 to 40	1 (5)	0	2 (11)	10 (14)
Mean age (sd)	29.4 (5.6)	27.2 (4.4)	29.5 (5.5)	28.8 (5.6)
Marital status				
Married/living with partner	16 (84)	16 (80)	18 (95)	58 (78)
Single/divorced	3 (16)	4 (20)	1 (5)	16 (22)
Education				
None	2 (11)	1 (5)	3 (16)	14 (19)
Primary	12 (63)	11 (55)	10 (53)	40 (54)
Secondary	5 (26)	7 (35)	6 (32)	19 (26)
Tertiary	0	1 (5)	0	0
Missing	0	0	0	1 (1)

FGD, focus group discussion.

**Table 3:** Demographic data for healthcare workers

	Healthcare workers *N*=43 *n* (%)
Age (in years)	
25 to 29	10 (23)
30 to 34	9 (21)
35 to 39	8 (19)
40 to 44	7 (16)
46+	9 (21)
Mean (sd)	38.1 (10.2)
Level of qualification	
ART clerk	3 (7)
Nurse	27 (63)
Clinical officer	6 (14)
Health surveillance	4 (9)
Medical assistant	2 (5)
Counsellor	1 (2)
Years in current position	
1 to 5	11 (26)
6 to 10	13 (30)
11 to 15	6 (14)
16 to 21	5 (12)
21+	8 (19)
Mean (sd)	12.4 (10.4)

ART, antiretroviral therapy.

Key study results are shown in Table 4.

**Table 4:** Key study findings

Acceptability of the drug regimen		
Perception of drug as health-enhancing	• Women reported feeling healthier on Option B+.	*It has given me strength, not getting sick often. Working like other people work and the body isn't weak*. (Lactating woman)
	• Women were motivated to initiate ART to regain their own health.	*Now I feel like my body is better than before because at first my body was very weak and I wasn't fat but I was very skinny*. (Lactating woman)
Normalization of appearance and infant feeding practice	• Women reported seeing positive results in the community, with sick women regaining their health.	*They* [the women] *say that the drugs have brought them back to their original shape because they are looking nice now*. (Pregnant woman)
	• Women reported that the healthy appearance of those on ART improved.	*I thought that if these people are taking the medicine and look like that, then why shouldn't I? So I decided to take the medicine*. (Lactating woman)
Scepticism about lifelong treatment	• Women struggled with the lifelong commitment to drugs.	*It was something difficult to accept because it was something you weren't expecting. You came here for ANC, they're testing your blood and they're telling you that today you have been found to be HIV+ and today you will have to start taking medicine. It was something that you weren't thinking about and you weren't expecting, so it was something difficult to accept, that “just today I should start taking medicine and I'll have to take the medicine for the rest of my life.”* (Pregnant woman)

**Acceptability of the PMTCT counselling services**		

Inappropriate timing of ART initiation	• HCWs and women expressed concerns about the counselling provided.	*The first counselling is not enough because it is not easy for someone to have come to ANC, have her blood tested, be found to be HIV positive and at the same time be told to initiate to ART. As a result more women just accept it to please us and* [so we will] *let them go, but when they go home they don't take the medicine or disclose to their husbands. So the programme of Option B+ is there but we don't provide enough information to women when they are to initiate ART, and it becomes a problem for them to follow the procedure*. (HCW)
	• Women were uncomfortable initiating ART the same day as learning their HIV status; they wanted to get their husband's permission first and receive a CD4 count before initiating ART.	*The woman cannot even start taking the medicine without the husband's knowledge and that's what makes women who come alone not take the decision to start taking the medicine*. (HCW)
	• HCWs and women reported not trusting the HIV test result and wanting to obtain an additional HIV test at a different facility for confirmation.	*I doubted* [the results] *very much, so that I went to four different hospitals, where I failed to get confirmation. At first I went to two private clinics, then the third time I went to Bottom Hospital, and the fourth time is when I went to Kapiri, and that's when I saw that these things are actually true*. (Pregnant woman)
Poor counselling procedures	• HCWs and women stressed the importance of adequate time being spent with the woman to educate her about ART and how to take care of herself.	*It is because he didn't have enough time to explain what we are supposed to be doing; he seemed to be rushing too when he was conducting his sessions, yet we needed enough time to learn what we are supposed to be doing*. (Lactating woman)
	• The most common recommendation was strengthening and extending the counselling.	*I would like the healthcare workers to have enough time to counsel their clients so that the clients can make good decisions*. (Pregnant woman)
Loss to follow-up	• HCWs discussed women not returning to the facility after initiation and the challenges of dealing with women who are lost to follow-up.	*Many clients are initiated on ART but a lot of them, when they go home and come back here, you'll find that they tell you that, “I haven't started taking the medicine,” or that, “I started taking them rather late and I've stopped taking them because of this and that reason,” and they are not coming back*. (HCW) *They come because of the pregnancy, but when* [the baby] *is delivered maybe we will not see her*. (HCW)

ANC, antenatal clinic; ART, antiretroviral treatment; HCW, healthcare worker; PMTCT, prevention of mother-to-child transmission.

### Acceptability of the drug regimen used in Option B+

#### Perception of drugs as health-enhancing

Women with prior receipt of antiretrovirals (ARVs) (e.g. for PMTCT in prior pregnancies) reported having fewer side effects and feeling healthier on Option B+ compared to their previous drug regimens. Women also reported increased appetite, weight gain and reduction in diarrhoea, vomiting, body sores, sickness and headaches. A pregnant woman stated, “I had some challenges with the old regimen because I was feeling headaches for some time but with this new regimen I am now okay.” Many women, both pregnant and postpartum, expressed a positive attitude towards Option B+ and discussed the importance of initiating ART to feel healthier, to prolong their life and have a HIV-negative child. “I was happy because I was told that I will be strong and will live longer” (postpartum woman).

#### Normalization of appearance

Both pregnant and postpartum women discussed seeing positive results in the community of women taking ART and looking healthy. Some women discussed positive encouragement from friends who were taking ART and were pleased with the results:

When I faced the problem [HIV diagnosis] and I asked other people, they said that, “aahh … we go and receive medicine and do you see the way we look right now.” And that's how I also accepted that I, too, should be receiving medicine so that I should look like my friends are looking. (Pregnant woman)

Women reported that identifying people who are HIV positive is difficult because those on medication look healthy. Some women reported that seeing these positive results in others encouraged them to start Option B+. A postpartum woman commented, “… for me I feel that the drugs are good because none can know that I am HIV positive.”

#### Scepticism about lifelong treatment

One of the main adherence challenges was continuing on ART after childbirth. The HCWs noted that many women did not see the need to take ART after giving birth. HCWs reported that some women asked if they were to stop taking ART when they finished breastfeeding and restart ART when they felt ill. The idea of continuing to take the medication as a lifelong commitment regardless of their health condition was a major concern to the both pregnant and postpartum women. “I was worried because I was thinking of taking drugs daily, [I] stopped [after] two weeks dosage … you don't feel like taking them any more … ” (postpartum woman). Another woman reported that the counselling had been sufficient but that she was “not ready to take the drugs for my whole life” (postpartum woman).

### Acceptability of PMTCT counselling services

#### ART initiation on the same day as HIV diagnosis (same-day initiation)

All groups discussed the challenges of asking women to initiate ART on the same day they learn their HIV status.

It's hard to convince them that that very same day they should start taking medicine and that when the viral load is high you are most likely to transmit the virus to the child; they don't really understand or that they should accept it. A lot of people live in denial. So it's too hard that even if you initiate them the same day at antenatal, it's not like when they go home they'll take the medicine; it's very difficult. (HCW)

Women were also uncomfortable initiating ART on the same day as learning their status, because they needed to get their husband's permission to initiate ART:

… here in Malawi most of the decision-making is done by the husband and the husband has remained behind and hasn't come along to the hospital. It becomes very difficult for the woman to make the decision alone to start taking the medicine in the absence of the husband. (HCW)

HCWs said it was more difficult for women to accept Option B+ without having their CD4 count, because they were familiar with the previous practice of testing the CD4 count to determine whether one should start ART. Some HCWs reported that convincing women to accept ART without a CD4 count was challenging. These women felt healthy and were reluctant to start ART:

For me to accept that I should start taking the medicine [was difficult] when I didn't even know what was my CD4 [count]. I was very argumentative. In my heart I was still uncomfortable that they don't know what my CD4 count is, and should I start taking medicine? (Pregnant woman)

Both pregnant and postpartum women reported feeling uncomfortable initiating ART because they did not believe the test result and wanted to get a second test (often at a different facility) before initiating ART. One HCW stated, “They don't believe in what they have been told, that they are HIV positive, and they refuse to take the medication.” Some women reported being too distracted with news of their HIV status to listen to or understand the counselling messages.

#### Poor counselling services

In general, both the pregnant and postpartum women felt that the HCWs were kind, friendly and supportive. However, both groups and the HCWs expressed concern about the counselling services, and the women felt that the way Option B+ was introduced was very important. The majority of women stressed the importance for HCWs to exercise patience and understanding when initiating a woman to Option B+, since this was crucial to acceptance and adherence of lifelong ART.

HCWs also agreed that it is very important for counselling to be thorough at the introduction of Option B+ and for the woman to be educated about ARVs so that she can make an informed decision:

Women can be advised that ARVs are not drugs for trial [to try]. When making a decision the person should know that it will be lifelong treatment. At the same time we need to highlight the challenges they will face when taking the drugs. Now if she understands, she will not get surprised if she [encounters] a problem; she will know that this is what the doctor said. (HCW)

Despite most women agreeing to start treatment, the HCWs worried that some women would not take the medication after leaving the facility:

The first counselling is not enough because it is not easy for someone to have come to ANC, have their blood tested, be found to be HIV positive, and at the same time be told to initiate ART. As a result more women just accept it to please us and [so we will] let them go, but when they go home they don't take the medicine and disclose to their husband. So the programme of Option B+ is there, but we don't provide enough information to women [about] when they are to initiate ART, and it becomes a problem for them to follow the procedure. (HCW)

The most common recommendation discussed by pregnant and postpartum women was strengthening and extending counselling. Some pregnant women reported that they were only given the drugs and told how to take them but not provided proper counselling. Some lactating women even claimed that they did not receive any counselling nor were they given any opportunity to ask questions, but were just given an appointment date to collect medicine. A pregnant woman explained, “I would like the HCWs to have enough time in counselling their clients so that the clients can be making good decisions.”

HCWs discussed how difficult it is to get some of the women to return to the facility to receive additional information on Option B+. The majority of the women reported they only received counselling once or twice, usually when they went for their initial HIV testing. A HCW stated, “… most of the time the clients [on Option B+] are on medication, yet they don't have detailed information on ARVs.”

Some postpartum women stated that they were able to get more comprehensive counselling when the HCWs visited them at their homes; however, women are provided with this option at some facilities and are free to decline. “When they [HCWs] started following me to my home … for further counselling, [that] is when I started getting good information about the ART” (postpartum woman). Both pregnant and postpartum women had many unanswered questions about Option B+, including taking it with other drugs, consequences of missing a pill, what to do if they did not plan to have more children and other topics.

#### Loss to follow-up

HCWs reported that some women did not return to the facility for counselling after receiving the first bottle of drugs and that the facilities were not able to track or retain women who were LTFU due to lack of transport, incentives for personnel and disclosure challenges. They were concerned that those who are LTFU will increase the number of people resistant to ART:

… many clients are initiating on ART but a lot of them when they go home and come back here, you'll find that they tell you that, “I haven't started taking the medicine” or that, “I started taking them rather late and I've stopped taking them because of this and that reason,” and they are not coming back. So we could have this problem in future whereby such people could develop resistance and for them to restart the medicine, they may not work properly. (HCW)

## Discussion

This study's results highlight the generally positive perception towards the drug regimen used in Option B+; women who had received ARVs for PMTCT in prior pregnancies experienced fewer side effects and women generally recognized the positive benefits of ART on their health. Women felt hopeful about prolonging their life and having an HIV-uninfected baby, yet still grappled with the fact that ART is a lifelong commitment. Many women struggled with initiating ART on the same day as learning their HIV status. Women wanted to discuss with their husbands first, receive a CD4 count and receive an HIV test at another facility to confirm their HIV status. HCWs expressed concern that women may just agree to take the drugs and then do as they please once they leave the facility. HCWs discussed concerns about women being LTFU and developing resistance.

Seeing the positive results of others encouraged women to initiate ART, and women emphasized the importance of restoring their health to return to their daily activities. The association of ART as “health enhancing” is a shift from the previous perception of ART being a sign of health deterioration. ART was previously seen as something that very “ill” patients living with HIV had to receive [8]. It is interesting to note that women discussed their own health more frequently than preventing transmission of HIV to their infant. The “normalization” of appearance is also a shift, as previous studies have documented ART, particularly when stavudine was a regimen component, being associated with a “deterioration” of appearance [9].

Our study also highlighted women's concern about commitment to lifelong treatment, which has also been documented in the literature [10]. This is an interesting contradiction expressed by the women in our findings, as women frequently mentioned the benefits of taking Option B+ for their own health benefits and yet discussed discontinuing ART after delivery or breastfeeding. This could be tied to the perception of ART being health enhancing and women believing they can take ART, recover their health and then stop taking ART.

Previous literature on short-course prophylaxis has documented similar feelings of hopelessness, shock and blaming oneself; however after the initial phase women seemed to accept their situation [11]. These results differed from our study findings, which did not find that acceptance increased after the initial phase.

One study in Malawi found that while time to ART initiation was significantly shorter, LTFU on ART increased from 5.8 to 11.2% after the introduction of Option B+ [12]. Higher attrition has been observed when women initiate ART to PMTCT during pregnancy compared with those who initiate for their own health [4]. Our study identified two main challenges to women accepting lifelong ART: the practice of initiating women on ART the same day they learn their HIV status and insufficient counselling to understand ART and the lifelong commitment of ART. Previous research has highlighted the challenges of same-day initiation of ART. A study on Option B+ in Malawi found that those who started ART on the day of HIV testing were almost twice as likely never to return to the clinic (adjusted OR 1.9; 95% CI 1.5 to 2.4) than Option B+ patients who started ART later (*p<*0.001) [4]. Our study findings show that women want more time to speak with their partner before initiating ART. Many women were surprised by their HIV diagnosis, questioned their test results and sought additional HIV testing elsewhere. Additionally, as CD4 count is perceived to be a main factor in determining whether treatment is necessary, many women found it difficult to accept initiating ART without it [13]. Additional counselling messages may need to be developed to explain why a CD4 count to establish ART eligibility is no longer required.

Our study found that some women felt that not enough time was available for counselling and encouraged a more thorough introduction to lifelong ART. It is interesting to note that similar findings were experienced with short-course ARV prophylaxis regarding service delivery. One systematic review found that the most common service delivery factors associated with low adherence included quality and timing of HIV testing and counselling; lack of emphasis of the importance of ARV adherence at post-counselling and follow-up care; and lack of male involvement, such as asking the partner to be tested or couples’ counselling. These service delivery challenges are similar to the concerns experienced under Option B+ [14]. HCWs expressed concern that women were not receiving adequate information. A study on Option B+ in Malawi found that facilities that offered additional adherence counselling beyond the required national guidelines had lower rates of early LTFU [4]. Other research articles have proposed evaluating new counselling models, such as sequenced counselling messages that first introduce ART as a PMTCT tool to protect the child and then introduce the benefits of lifelong ART and prepare women for long-term adherence and retention [15].

There are limitations to this study. The study population has limitations, as it only included women who accessed the health facility, initiated ART, had a live child and breastfed their child. This population does not include more vulnerable populations who may not have access to a facility, rejected Option B+, whose child did not live or who were unable to breastfeed their child. Participants were 18 years of age and older; thus the views of younger women have not been explored.

## Conclusions

Although Option B+ has significantly increased the number of women initiating ART, there are still challenges that need to be addressed to strengthen initiation, adherence and retention in the Option B+ programme; same-day ART initiation stands out as particularly challenging. Strategies to strengthen the counselling services at initiation need to be developed to improve same-day ART initiation and long-term ART adherence. More research evaluating different counselling models and messages for same-day initiation ART is needed. These are important lessons learned for Malawi and other countries implementing Option B+ as they further develop counselling messages and patient education services.
